# Supplementary material for: Optimizing cabin air inlet velocities and personal risk assessment: Introducing the Personal Contamination Ratio (PCR) method for enhanced aircraft cabin infection risk evaluation
Source: PLoS One. 2024 Sep 6;19(9):e0309730. doi: 10.1371/journal.pone.0309730 (PMC11379313; doi:10.1371/journal.pone.0309730)
Supplement: S1 Data — (DOCX) [file pone.0309730.s001.docx]

**S1 Data for Figure 2**

| 2.6M | | 3.92M | | 5.2M | | 6.51M | | 7.84M | |
| --- | --- | --- | --- | --- | --- | --- | --- | --- | --- |
| 0.00 | 0.26 | 0.00 | 0.25 | 0.00 | 0.24 | 0.00 | 0.26 | 0.00 | 0.26 |
| 1.00 | 0.13 | 1.00 | 0.14 | 1.00 | 0.18 | 1.00 | 0.12 | 1.00 | 0.13 |
| 2.00 | 0.03 | 2.00 | 0.03 | 2.00 | 0.04 | 2.00 | 0.03 | 2.00 | 0.03 |
| 3.00 | 0.01 | 3.00 | 0.02 | 3.00 | 0.02 | 3.00 | 0.02 | 3.00 | 0.02 |
| 4.00 | 0.01 | 4.00 | 0.02 | 4.00 | 0.03 | 4.00 | 0.02 | 4.00 | 0.02 |
| 5.00 | 0.02 | 5.00 | 0.03 | 5.00 | 0.02 | 5.00 | 0.02 | 5.00 | 0.02 |
| 6.00 | 0.01 | 6.00 | 0.03 | 6.00 | 0.02 | 6.00 | 0.02 | 6.00 | 0.02 |
| 7.00 | 0.03 | 7.00 | 0.02 | 7.00 | 0.02 | 7.00 | 0.02 | 7.00 | 0.02 |
| 8.00 | 0.01 | 8.00 | 0.02 | 8.00 | 0.01 | 8.00 | 0.02 | 8.00 | 0.02 |
| 9.00 | 0.01 | 9.00 | 0.01 | 9.00 | 0.01 | 9.00 | 0.01 | 9.00 | 0.01 |
