# Supplementary material for: Optimizing cabin air inlet velocities and personal risk assessment: Introducing the Personal Contamination Ratio (PCR) method for enhanced aircraft cabin infection risk evaluation
Source: PLoS One. 2024 Sep 6;19(9):e0309730. doi: 10.1371/journal.pone.0309730 (PMC11379313; doi:10.1371/journal.pone.0309730)
Supplement: S2 Data — (DOCX) [file pone.0309730.s002.docx]

**S2 Data for Figure 5**

| Series 1 at /LINE:Line A |  | literature |  |  |
| --- | --- | --- | --- | --- |
| Velocity [ m s^-1 ] | Y [ m ] | Velocity [ m s^-1 ] | Y [ m ] | X [ m ] |
| 0.035263 | 0.8 | —————— | ————— | ————— |
| 0.0348 | 0.877778 | —————— | ————— | ————— |
| 0.035129 | 0.955556 | 0.095649 | 0.98262 | -1.262 |
| 0.03947 | 1.033333 | —————— | ————— | ————— |
| 0.069114 | 1.111111 | 0.1033 | 1.0987 | -1.3549 |
| 0.263716 | 1.188889 | 0.14687 | 1.2032 | -1.3084 |
| 0.417902 | 1.266667 | —————— | ————— | ————— |
| 0.442293 | 1.344444 | 0.26295 | 1.3194 | -0.96008 |
| 0.446431 | 1.422222 | 0.43735 | 1.4471 | -1.32 |
| 0.257461 | 1.5 | 0.2501 | 1.5168 | -1.2852 |

| Series 2 at /LINE:Line F |  | literature |  |  |
| --- | --- | --- | --- | --- |
| Velocity [ m s^-1 ] | Y [ m ] | Velocity [ m s^-1 ] | Y [ m ] | X [ m ] |
| 0.031795 | 0.8 | —————— | ————— | ————— |
| 0.034604 | 0.877778 | —————— | ————— | ————— |
| 0.042754 | 0.955556 | 0.066577 | 0.99423 | 1.3473 |
| 0.046093 | 1.033333 | —————— | ————— | ————— |
| 0.106543 | 1.111111 | 0.10041 | 1.1104 | 1.1383 |
| 0.276332 | 1.188889 | 0.13958 | 1.2149 | 1.2428 |
| 0.420037 | 1.266667 | 0.30079 | 1.3078 | 1.0106 |
| 0.445859 | 1.344444 | —————— | ————— | ————— |
| 0.450812 | 1.422222 | 0.45022 | 1.4239 | 1.2544 |
| 0.194368 | 1.5 | 0.19035 | 1.5168 | 1.3473 |

| Series 3 at /LINE:Line B |  | literature |  |  |
| --- | --- | --- | --- | --- |
| Velocity [ m s^-1 ] | Y [ m ] | Velocity [ m s^-1 ] | Y [ m ] | X [ m ] |
| 0.052302 | 0.8 | —————— | ————— | ————— |
| 0.058281 | 0.877778 | —————— | ————— | ————— |
| 0.060638 | 0.955556 | 0.059789 | 0.98262 | -0.90202 |
| 0.060082 | 1.033333 | 0.059622 | 1.0407 | -0.93685 |
| 0.057036 | 1.111111 | 0.055327 | 1.1104 | -1.0297 |
| 0.051574 | 1.188889 | 0.050635 | 1.1916 | -0.85557 |
| 0.042224 | 1.266667 | 0.04115 | 1.2729 | -0.83234 |
| 0.034005 | 1.344444 | 0.063111 | 1.3426 | -1.1226 |
| 0.022703 | 1.422222 | 0.12404 | 1.4123 | -1.1923 |
| 0.00793 | 1.5 | —————— | ————— | ————— |

| Series 4 at /LINE:Line E |  | literature |  |  |
| --- | --- | --- | --- | --- |
| Velocity [ m s^-1 ] | Y [ m ] | Velocity [ m s^-1 ] | Y [ m ] | X [ m ] |
| 0.051546 | 0.8 | —————— | ————— | ————— |
| 0.060093 | 0.877778 | —————— | ————— | ————— |
| 0.064484 | 0.955556 | 0.065169 | 0.98262 | 0.89443 |
| 0.064343 | 1.033333 | 0.064164 | 1.0407 | 0.91766 |
| 0.061064 | 1.111111 | 0.061206 | 1.1104 | 0.95249 |
| 0.054997 | 1.188889 | 0.053697 | 1.18 | 0.98733 |
| 0.045452 | 1.266667 | 0.043822 | 1.2613 | 0.94088 |
| 0.028454 | 1.344444 | 0.028029 | 1.3542 | 0.94088 |
| 0.014384 | 1.422222 | 0.064969 | 1.4123 | 1.0454 |
| 0.017499 | 1.5 | —————— | ————— | ————— |

| Series 5 at /LINE:Line C |  | literature |  |  |
| --- | --- | --- | --- | --- |
| Velocity [ m s^-1 ] | Y [ m ] | Velocity [ m s^-1 ] | Y [ m ] | X [ m ] |
| 0.048867 | 0.8 | —————— | ————— | ————— |
| 0.050111 | 0.877778 | —————— | ————— | ————— |
| 0.049483 | 0.955556 | 0.05108 | 0.98262 | -0.61172 |
| 0.046377 | 1.033333 | 0.047689 | 1.0407 | -0.61172 |
| 0.041004 | 1.111111 | 0.041449 | 1.1104 | -0.63494 |
| 0.035938 | 1.188889 | 0.034859 | 1.18 | -0.54205 |
| 0.030305 | 1.266667 | 0.052494 | 1.2613 | -0.693 |
| 0.024056 | 1.344444 | 0.17319 | 1.331 | -0.5885 |
| 0.016236 | 1.422222 | 0.18116 | 1.4703 | -0.20119 |
| 0.010535 | 1.5 | —————— | ————— | ————— |

| Series 6 at /LINE:Line D |  | literature |  |  |
| --- | --- | --- | --- | --- |
| Velocity [ m s^-1 ] | Y [ m ] | Velocity [ m s^-1 ] | Y [ m ] | X [ m ] |
| 0.060777 | 0.8 | —————— | ————— | ————— |
| 0.06466 | 0.877778 | —————— | ————— | ————— |
| 0.059758 | 0.955556 | 0.11961 | 0.98262 | 0.52285 |
| 0.051919 | 1.033333 | 0.11902 | 1.0407 | 0.54607 |
| 0.043079 | 1.111111 | 0.10606 | 1.122 | 0.58091 |
| 0.035266 | 1.188889 | 0.087319 | 1.1916 | 0.59252 |
| 0.02897 | 1.266667 | 0.086641 | 1.2729 | 0.32134 |
| 0.023174 | 1.344444 | 0.049226 | 1.3542 | 0.30973 |
| 0.014022 | 1.422222 | 0.015989 | 1.4239 | 0.27489 |
| 0.018275 | 1.5 | —————— | ————— | ————— |

| Series 7 at /LINE:Line aisle |  | literature |  |  |
| --- | --- | --- | --- | --- |
| Velocity [ m s^-1 ] | Y [ m ] | Velocity [ m s^-1 ] | Y [ m ] | X [ m ] |
| 0.034325 | 1.1 | —————— | ————— | ————— |
| 0.020656 | 1.2 | —————— | ————— | ————— |
| 0.01038 | 1.3 | —————— | ————— | ————— |
| 0.003214 | 1.4 | 0.058499 | 1.4006 | 0.0078196 |
| 0.006677 | 1.5 | 0.006179 | 1.4935 | 0.10071 |
| 0.02065 | 1.6 | 0.020334 | 1.6097 | 0.089103 |
| 0.01564 | 1.7 | 0.047158 | 1.6952 | -0.18958 |
| 0.005659 | 1.8 | 0.026322 | 1.7997 | -0.18958 |
| 0.005481 | 1.9 | 0.022791 | 1.9042 | -0.038628 |
| 0.006876 | 2 | 0.01638 | 2.0087 | 0.10071 |
