# Supplementary material for: Optimizing cabin air inlet velocities and personal risk assessment: Introducing the Personal Contamination Ratio (PCR) method for enhanced aircraft cabin infection risk evaluation
Source: PLoS One. 2024 Sep 6;19(9):e0309730. doi: 10.1371/journal.pone.0309730 (PMC11379313; doi:10.1371/journal.pone.0309730)
Supplement: S3 Data — (DOCX) [file pone.0309730.s003.docx]

**S3 Data for Figure 13**

| **0.51m/s** | **A** | **B** | **C** | **D** | **E** | **F** |
| --- | --- | --- | --- | --- | --- | --- |
| **ROW1** | 0 | 0 | 0 | 0 | 0 | 0 |
| **ROW2** | 0 | 0 | 0 | 0 | 0 | 0 |
| **ROW3** | 0 | 0 | 0 | 0 | 0 | 0 |
| **ROW4** | 0 | 0 | 0 | **0.17** | 0 | **0.17** |
| **ROW5** | **0.32** | **0.32** | **1** | 0 | 0 | 0 |
| **ROW6** | **0.99** | **1** | **0.99** | **0.87** | **0.74** | **0.17** |
| **ROW7** | **0.92** | **1** | **0.99** | **0.96** | **0.85** | **0.68** |
| **ROW8** | **0.61** | **0.96** | **0.92** | **0.74** | **0.78** | **0.82** |
| **ROW9** | **0.61** | **0.61** | **0.68** | **0.53** | **0.68** | **0.68** |

| **0.77m/s** | **A** | **B** | **C** | **D** | **E** | **F** |
| --- | --- | --- | --- | --- | --- | --- |
| **ROW1** | 0 | 0 | 0 | 0 | 0 | 0 |
| **ROW2** | 0 | 0 | 0 | 0 | 0 | 0 |
| **ROW3** | 0 | 0 | 0 | 0 | 0 | 0 |
| **ROW4** | **0.34** | **0.18** | **0.71** | 0 | 0 | 0 |
| **ROW5** | 0 | 0 | **1** | **0.65** | **0.56** | **0.99** |
| **ROW6** | **0.65** | **0.46** | **0.46** | **0.99** | **0.92** | **1** |
| **ROW7** | **0.96** | 0 | **0.71** | **0.9** | 0 | 0 |
| **ROW8** | **0.99** | **0.76** | **0.85** | **0.71** | **0.92** | **1** |
| **ROW9** | **0.18** | **0.18** | 0 | 0 | 0 | **0.85** |

| **1.03m/s** | **A** | **B** | **C** | **D** | **E** | **F** |
| --- | --- | --- | --- | --- | --- | --- |
| **ROW1** | 0 | 0 | 0 | 0 | 0 | 0 |
| **ROW2** | 0 | 0 | 0 | 0 | 0 | 0 |
| **ROW3** | 0 | 0 | 0 | 0 | 0 | 0 |
| **ROW4** | 0 | 0 | **0.19** | 0 | 0 | 0 |
| **ROW5** | 0 | 0 | **1** | 0 | 0 | 0 |
| **ROW6** | 0 | **0.19** | 0 | 0 | 0 | 0 |
| **ROW7** | 0 | **0.65** | **0.71** | **0.19** | **0.47** | 0 |
| **ROW8** | 0 | **0.99** | **1** | **0.98** | **0.34** | 0 |
| **ROW9** | 0 | **1** | **1** | **0.57** | 0 | 0 |
