# Supplementary material for: Optimizing cabin air inlet velocities and personal risk assessment: Introducing the Personal Contamination Ratio (PCR) method for enhanced aircraft cabin infection risk evaluation
Source: PLoS One. 2024 Sep 6;19(9):e0309730. doi: 10.1371/journal.pone.0309730 (PMC11379313; doi:10.1371/journal.pone.0309730)
Supplement: Attachment — Submitted filename: Response to Reviewers.docx [file pone.0309730.s004.docx]

**Response to Reviewer #1's Comments**

**Ref:** PONE-D-24-00989

**Title:** Optimizing cabin air inlet velocities and personal risk assessment: introducing the Personal Contamination Ratio (PCR) method for enhanced aircraft cabin infection risk evaluation

We're truly grateful for the reviewer's supportive feedback, which means a lot to us.

**Response to Reviewer #2's Comments**

**Ref:** PONE-D-24-00989

**Title:** Optimizing cabin air inlet velocities and personal risk assessment: introducing the Personal Contamination Ratio (PCR) method for enhanced aircraft cabin infection risk evaluation

**Comments:**

1. The Authors present the analysis of the breathing zone as a strong point, but the dynamics of breathing and particle inhalation are completely neglected. These aspects considerably alters the distribution of particles in the breathing zone, making the results obtained unreliable.

We thank the reviewers for raising the insightful comments. The authors would like to clarify the major objective of this study is to propose a novel holistic infection risk assessment framework by incorporating the concept of the breathing zone and the Personal Contamination Ratio (PCR) into the original Wells-Riley model. Based on the detailed spatial-temporal information of the tracked droplets from the CFD simulations, it allowed us to obtain a more reasonable infection risk quantification considering the particle distribution.

We acknowledged the significance of considering dynamics related to breathing and particle inhalation, which was believed to enhance the overall robustness of the assessment. While those aspects were believed to be beneficial, including them into the simulation was expected to significantly improve the overall computational cost. Considering the complex transient nature of the breathing patterns (boundary condition variations), and multi-scale (from cabin environment, m^3^, to human nasal, mm^3^) and multi-coupling challenges, further including those setups within a densely-occupied cabin space was expected to exponentially increase the simulation difficulty and time. Moreover, many existing studies overlook the influence of the breathing pattern, as exemplified by the following instances:

*[1] Kong B, Zou Y, Cheng M, Shi H, Jiang YJAS. Droplets transmission mechanism in a commercial wide-body aircraft cabin. 2022;12(10):4889.*

*[2] Wang F, Zhang TT, You R, Chen Q. Evaluation of infection probability of Covid-19 in different types of airliner cabins. Build Environ. 2023;234:110159.*

*[3] You R, Lin CH, Wei D, Chen QJIA. Evaluating the commercial airliner cabin environment with different air distribution systems. 2019;29(5):840-53.*

*[4] Choi E-S, Yook S-J, Kim M, Park DJT. Study on the Ventilation Method to Maintain the PM10 Concentration in a Subway Cabin below 35 μg/m3. 2022;10(10):560.*

*[5] Zhang M, Yu N, Zhang Y, Zhang X, Cui YJP. Numerical simulation of the novel coronavirus spread in commercial aircraft cabin. 2021;9(9):1601.*

2. English language needs improvement. At some points, the sentence construction is convoluted; contracted forms are also present, which are not suitable for a scientific paper.

Thanks for raising this issue which we agree with. We checked the grammar and wording in the manuscript and revised them accordingly, as follows:

1. Page 2, lines 39-40. ‘Air cabins play an important role in spreading infectious diseases (8-11).’
2. Page 3, lines 63-64. ‘However, Yan, Li (18)'s investigations predominantly focused on droplets of a representative size, which may not be widely applicable to all scenarios.’
3. Page 10, lines 281-283. ‘Compared to previous methods used for predicting droplet spread, this study introduces a new exposure risk quantification method which named $PCR$ ($Personal Contamination Ratio$).’
4. Page 12, lines 347-348. ‘Consequently, this research begins with visualizing cabin airflow characteristics to better understanding of the particle distribution.’
5. Page 13, lines 374-375. ‘The distribution of particle in the cabin ~~is~~ influenced not only by inlet air velocity but also ~~by~~ the direction of airflow.’
6. Page 17, lines 488-489. ‘Their trajectories are altered significantly in response to a substantial increase in inlet air velocity.’

3. The description of the simulated scenario is confusing. It is stated that 6 people have been simulated (line 152), but in Figure 1 more passengers are depicted. In addition, authors should demonstrate that the simulated domain (restricted to a section of the whole cabin) is representative of the problem under investigation.

Thanks for the reviewer’s comment. The authors would like to clarify that in this study, a total of 18 passengers were modelled within an aircraft cabin. 6 passengers were constructed in a single row with one aisle to mimic the real cabin layout. Based on the review’s comment, the authors found that the previous description of the model can be quite confusing for the readers and such description has now been revised based on the reviewer’s comment (please see page6, lines 154-155).

‘As shown in Figure 1, passengers and seats are arranged on two sides of the cabin. There are three rows, each accommodating six persons, making a total number of 18 passengers.’

As for the second concern, the authors acknowledged that investigating the in-cabin aerosol transmission scenario with a full-scale cabin model would be perfect as it provides a comprehensive picture of the particle transport and distribution characteristics. However, the authors would like to point out that restoring such phenomena can be really time-consuming (e.g. solving the large-scale, multi-phase simulation). In this study, the authors adopted the aforementioned layout as recent studies demonstrate that severe transmission generally occurs longitudinally, within the 3 rows *[1]*. Most existing studies are based on such outcomes to model the in-cabin transmission scenarios in recent years *[2-5]*. The authors acknowledge that the current cabin model would not be perfect, while based on the existing findings and mainstream studies, such a computational model was expected to be reasonable and representative. Additionally, we have added some text in page 5, lines 137-139. Thanks for the reviewer’s comment.

‘Building upon previous literature (18, 26, 27, 34, 35), to optimize computational resources, we have chosen to focus our analysis on a representative subset of the cabin section with three rows.’

*[1] Silcott, David, et al. "TRANSCOM/AMC commercial aircraft cabin aerosol dispersion tests." (2020).*

*[2] Zhao, Yingjie, et al. "Numerical simulation study on air quality in aircraft cabins." Journal of Environmental Sciences 56 (2017): 52-61.*

*[3] Zee, Malia, et al. “Computational fluid dynamics modeling of cough transport in an aircraft cabin.” Scientific reports 11.1 (2021): 23329.*

*[4] Kong, Benben, et al. “Droplets transmission mechanism in a commercial wide-body aircraft cabin.” Applied Sciences 12.10 (2022): 4889.*

*[5] Yan, Yihuan, et al. "Evaluation of cough-jet effects on the transport characteristics of respiratory-induced contaminants in airline passengers’ local environments." Building and environment 183 (2020): 107206.*

4. Authors should specify the software employed to carry out CFD simulations.

We appreciate the reviewer for reminding this issue. We have added specific details regarding the software used for the CFD simulations on page 8, lines 222-223.

‘This study utilized the commercial computational fluid dynamics (CFD) software ANSYS Fluent 2021 R1 for conducting all numerical calculations.’

5. It is not sufficient to state that “Building upon existing research findings, our simulations employed a mesh grid totaling 6.51 million polyhedral cells within the cross-section of the three-row cabin” (lines 152-154). Unless the authors have carried out previous studies on the same cabin model, a grid sensitivity analysis must be performed.

We appreciate the reviewer for bringing up this issue. We acknowledge the importance of conducting mesh sensitivity analysis for mesh generation, and we have provided the information about the mesh sensitivity analysis below. The horizontal axis of the table represents velocity, while the vertical axis represents the length of the validation line. Through the comparison from this picture, there is no considerable deviation in the velocity field noticed after increasing the number of mesh elements from 6.51 million to 7.84 million. Thus, we chose to use a mesh setup with 6.51 million elements for the simulations. Additionally, in the manuscript on page 6, lines 155-162 and 179-180, we have included a description of the mesh independence test as follow:

‘To achieve mesh independence, this study experimented with five sets of mesh configurations, utilizing total mesh elements of 2.60 million, 3.92 million, 5.20 million, 6.51 million, 7.84 million, respectively. Significant discrepancies in velocity were observed among the groups with grid numbers of 2.60, 3.92, and 5.20 million, while an increase in mesh elements from 6.51 million to 7.84 million revealed negligible deviation in the velocity field, as illustrated in Figure 3. This indicates that a mesh of 6.51 million elements is suitable for simulating airflow within the computer cabin. Therefore, in our simulations employed a mesh grid totaling 6.51 million polyhedral cells within the cross-section of the three-row cabin, achieving a maximum skewness of 0.81.’

6. A complete description of the boundary conditions should be provided. The periodic boundary condition set for the “periodic faces” needs to be discussed further.

We agree with the constructive suggestion. The detailed description of the periodic boundary condition is added to page 7, line 200.

‘…we have set the floor, seats, walls, and ceiling as no-slip surfaces. Additionally, we employed translation as the periodic face type.’

7. The governing equations presented in section 2.4 (Equation 1 and 2) are for a laminar, incompressible and unsteady flow. These are not the governing equations of the problem under investigation. The URANS equations must be provided.

In addition, the Boussinesq approximation seems to be used to model the effects of buoyancy (by considering the density constant in the transient and convective terms), but then in the gravitational term its dependence on temperature is not considered.

Thanks for raising this issue. We have revised the formula, with specific modifications outlined on page 8, lines 226-233, as follows:

$$\begin{aligned} \left( \vec{v} \right.\cdot\left. \nabla\right)\vec{v}=-\frac{\nabla P}{\rho}+\frac{\mu}{\rho}\nabla^{2}\vec{v}+\vec{g}\#\left（ 2 \right） \end{aligned}$$

$$\begin{aligned} \rho C_{p}\vec{u}\cdot\nabla T=k\nabla^{2}T+\rho C_{p}\vec{g}\cdot\vec{u}+Q\#\left（ 3 \right） \end{aligned}$$

‘$C_{p}$ is the Specific heat capacity at constant pressure. $T$ is temperature. And $k$ is the thermal conductivity coefficient. $Q$ is the heat source term.’

$$\begin{aligned} \rho\bar{v}_{i}\frac{\partial\bar{\Phi}}{\partial x_{i}}-\frac{\partial}{\partial x_{i}}\left[ \Gamma_{(\varphi,eff)}\frac{\partial\bar{\Phi}}{\partial x_{i}} \right]=S_{\varphi}\#\left( 4 \right) \end{aligned}$$

‘Where $\bar{u}_{i}$ represents the components of fluid velocity, $\Gamma_{\varphi,eff}$ is the effective diffusion coefficient, $\varphi$ is the flow variables, and $S_{\varphi}$ represents the source term.’

Additionally, we sincerely apologize for any confusion caused to readers due to our oversight. The misleading descriptions in the manuscript have now been corrected, as evidenced on page 8, lines 223-224 as follows:

‘The Navier-Stokes(N-S) equations with the Boussinesq approximation were used to simulate airflow field in Eulerian method’

8. The drag force (equation 3) should be evaluated as a function of the droplet Reynolds number.

Thanks for your reminder. The Reynolds number is already included in the formula. To enhance the clarity and conciseness of the formula expression, the first term on the right-hand side of the equation is separated and presented individually.

$$\frac{d\vec{u_{p}}}{dt}\boldsymbol{=}\frac{18\mu}{\rho_{p}d_{p}^{2}}\left( \vec{u}-\vec{u_{p}} \right)+\frac{\vec{g}\left( \rho_{p}-\rho\right)}{\rho_{p}}$$

9. The scenario analyzed for particle emission is not realistic at all. It should also be described in more detail, not simply reporting the total number of particles emitted.

The diameters (1 and 5 µm) are not representative of a real scenario, nor are the velocity (fixed at 1 m/s) and the direction of release.

Thank you for bringing up this question. We would like to clarify that the details of the particle information can be found in Figure 1, specifying both the location and angle of incidence. Additionally, we have provided further details regarding the particle emission location on pages 5, lines 146-151. The specifics are as follow.

In consideration of the velocity and direction of release, we carefully referred to peer-reviewed literature and set up the specifics based on a scenario involving a stationary seated individual in a steady state, as outlined in *reference [3-4]*. We believe this adequately explains the information regarding the particle inlet.

‘Following the recommendations of Kong, Zou (26), we have represented the CSP model's particle injection, located on the face, as a simplified circular inlet with a diameter of 1.24 cm. In accordance with findings from Haselton and Sperandio (36) and Kuga, Wargocki (28), the exhalation zone is identified to span from 27° and 33°. This study set a central value of 30° as the particle injection angle.’

As for the particle diameter, we have provided references as follow to support the significance of investigating diameters of 1 and 5 µm. In particular, the *reference - [5]* illustrates that the particle diameter of human respiratory particles is almost below 8 µm:

*[1] Thomas, Richard James. "Particle size and pathogenicity in the respiratory tract." Virulence 4.8 (2013): 847-858.*

*[2] Shang, Y. D., K. Inthavong, and J. Y. Tu. "Detailed micro-particle deposition patterns in the human nasal cavity influenced by the breathing zone." Computers & Fluids 114 (2015): 141-150.*

*[3] Kuga K, Wargocki P, Ito KJIa. Breathing zone and exhaled air re‐inhalation rate under transient conditions assessed with a computer‐simulated person. 2022;32(2):e13003.*

*[4] Zhang M, Yu N, Zhang Y, Zhang X, Cui YJP. Numerical simulation of the novel coronavirus spread in commercial aircraft cabin. 2021;9(9):1601.*

*[5] Zhang, Hualing, et al. "Documentary research of human respiratory droplet characteristics." Procedia engineering 121 (2015): 1365-1374.*

10. How would the model proposed by the Authors improve the Wells-Riley model, by changing the perspective from global to local (section 2.5)?

In the Referee’s opinion, the proposed model is not reliable to provide quantitative information about the risk of infection; rather, it gives information about the relative weight between different zones. For this purpose, it would have been sufficient to show the concentration of particles in different zones.

Thank you for your insightful question and feedback. The approach introduced in our study offers a significant departure from the global perspective typically employed in prior research. By modifying the methodology for calculating exposure risk (as highlight in the formula) and integrating the PCR factor into the infection risk calculation, we shift the focus from a global outlook to an individualized perspective. This shift enables us to accurately quantify the localised infection risk of each seated passenger rather than analysing the global infection risk, thereby enhancing the reliability of the quantitative information provided.

As for the second concern, we would like to clarify that the method employed in our study, the Eula-Laglang method, is specially tailored to analyze particle trajectory paths. Our primary objective is to comprehensively investigate particle trajectories from infection source to each individual's breathing zone. Within this framework, we have quantified particle numbers within each breathing zone to assess infection and exposure risks across different areas. Besides, we have revised the calculation formula for Possibility of Infection to ensure a more accurate characterization of each individual’s PI value.

**The way to improve the Wells-Riley model in this study:**

As for the improvement to the Wells-Riley model, we specifically adjust the calculation of the personal contamination ratio by shifting from considering the total particle amount in a zone to evaluating the particle concentration within each individual's breathing zone.

$$Personal Contamination Ratio=\frac{N_{vs}\left( V \right)}{N_{vr}(V)}$$

$$\begin{aligned} =\frac{\sum_{i(single respirable)}V_{cuboid-i}\times C}{V_{cuboid-i}\times C}=\frac{\sum_{i(single respirable)}V_{cuboid-i}}{V_{cuboid-i}\times C} \end{aligned}$$

Among them, $N_{vs}\left( V \right)$ indicates the cumulative number of droplets in a single breathing zone, and $N_{vr}(V)$ represents the cumulative number of droplets particles in all breathing zones. $C$ represents the droplets concentration in the droplets discharged at the moment of discharge, and $d_{i}$ is the original diameter of the $i$ th expelled droplet.

$$\begin{aligned} PI=1-exp\left( -\frac{ln2}{\theta{TCID}_{50}}\cdot PCR\cdot N_{particles}\cdot p\cdot t \right) \end{aligned}$$

Among them, $\theta$ denotes the ratio coefficient of ${HID}_{50}$ (median infective dose in humans) to ${TCID}_{50}$, specifically we utilize the influenza data for estimation. $N_{particles}$ represents the number of the particles, $p$ signifies the pulmonary ventilation rate, and $t$ represents the duration of the flight.

11. What is the error between PIV measurements and CFD results? The agreement seems to be very poor, especially for lines A and F (Figure 4). Such disagreement raises questions regarding the turbulence model, the boundary conditions and the grid sensitivity analysis.

In addition, the section of the experimental-numerical comparison should be highlighted in the computational domain and the scenario considered for the validation should be described.

Thank you for raising this question. In the research field of aircraft cabin flow fields, there are various factors influencing the flow field, which further lead to the differences observed between PIV measurements and CFD results. For example, the CSP model used in the experiment differs entirely from the CSP model employed in our simulation, which may result in difference between the two.

Regarding Lines A and F as depicted in Figure 4, although there are numerical variations compared to PIV measurements, both lines exhibit consistent trends. Such circumstances are widely accepted in the research field and can also be considered as demonstrating reliable consistency. It's essential to note that our simulation employs a different CSP model compared to the experimental setup, which may contribute to these variations. Similar cases have also been observed in other peer-reviewed articles, as follows:

*[1] Yan, Yihuan, et al. "Evaluation of airborne disease infection risks in an airliner cabin using the Lagrangian-based Wells-Riley approach." Building and environment 121 (2017): 79-92.*

12. I find the representation in Figure 5 of little use, with the experimental vectors barely visible. The legend is also missing (as in the later images). It would be more useful to represent the entire measured velocity field.

In general, the representation with vectors is unreadable and does not allow to visualize the presence of recirculation zones; in this sense, streamlines would be more suitable.

Thanks for your reminder. We’ve addressed the issue about the legend of Figure 5. Figure 5 serves to validate our research model by comparing its speed characteristics with those documented in previous literature. It demonstrates that our model closely aligns with the velocity flow field observed in experimental models, affirming its ability to simulate indoor air in the cabin effectively. While we acknowledge that streamlines might provide a more visually striking presentation, we believe that employing velocity vector arrows is more appropriate here. This method is more concise and clearer, facilitating a direct comparison between simulated and experimental velocity directions. To enhance the efficiency of comparing experimental and simulated vector directions, we further modified the images by appropriately magnifying the arrow sizes and adjusting the visuals accordingly. This was done to facilitate readers' accurate comprehension of the effectiveness of our model while reading. At the same time, we have included the literature sources pertaining to the raw experimental data on page 12, lines 340-341.

‘The vector raw experimental data is sourced from Li, Cao (16).’

13. A picture depicting particle spatial distribution should be provided, commented with reference to the simulated velocity fields.

Thank you for your guidance. In this paper, trajectories are employed to visually represent particle movement, while also indirectly demonstrating the spatial distribution of particle concentration through the computation of infection risk and exposure risk, especially, the infection risk also reflects the relative pathogen concentration distribution. We believe that Figure 10 adequately demonstrates the particle distribution, particularly through the visualization of particle trajectories in the streamline plot, which clearly depicts particle movement and residence time. Regarding particle velocity, we believe its analysis offers limited help in understanding particle spread within the air cabin.

14. A strange effect is present in the velocity fields of the simulated scenarios whereby the air jet is strongly drawn back to the wall, despite being released at a certain angle toward the inside of the cabin. This effect is not present in the validation scenario and should be explained by the authors.

Thank you for highlighting this observation. Indeed, we have noted peculiar behavior in the velocity field during the simulation process. It appears that the airflow tends to move towards the rear of the cabin. Similar occurrences can be found in literature as follow, whether through experimental observations or computational simulations, perhaps due to the specific configuration of the cabin. Referencing these studies and through careful analysis, we have found that this complex phenomenon is evidently influenced by numerous factors, such as differences in the internal structure of the cabin and the direction of incoming airflow. Therefore, the airflow moving towards the rear of the cabin can be considered as a common phenomenon.

*[1] Yan, Yihuan, et al. "Evaluation of airborne disease infection risks in an airliner cabin using the Lagrangian-based Wells-Riley approach." Building and environment 121 (2017): 79-92.*

15. How is it possible for air to be completely carried behind (resulting in infection only for those passengers sitting behind the infected person)? The explanation provided by the authors (lines 379-383) is not convincing and the effect of the boundary conditions set at the "periodic faces" should be investigated.

Thank you for bringing up this question. The mechanism by which air is predominantly carried behind passengers seated behind an infected individual is multifaceted, influenced by factors such as the angle of air inlet and the configuration of the cabin seating plan. Therefore, we perceive this phenomenon as a plausible occurrence within the simulation context. Similar observations have been documented in other peer-reviewed literature *[1-4]*. Besides, we maintain that the presence of "periodic faces" does not compromise the accuracy of our findings.

*[1] Yan, Yihuan, et al. "Evaluation of airborne disease infection risks in an airliner cabin using the Lagrangian-based Wells-Riley approach." Building and environment 121 (2017): 79-92.*

*[2] Kong, Benben, et al. "Droplets transmission mechanism in a commercial wide-body aircraft cabin." Applied Sciences 12.10 (2022): 4889.*

*[3] Silcott, David, et al. "TRANSCOM/AMC commercial aircraft cabin aerosol dispersion tests." (2020).*

*[4] Zhao, Yingjie, et al. "Numerical simulation study on air quality in aircraft cabins." Journal of Environmental Sciences 56 (2017): 52-61.*

16. Representing the possibility of infection for the source (Figure 12) makes no sense. In addition, there is probably a typo in the legend (the highest probability is equal to 0.25%, which is very low).

Thank you for raising this question. We apologize for any mistakes in the description of the Figure 13 (formerly Figure 12). Maintaining the representation of infection possibility for the source in Figure 13 is deemed necessary, as it illustrates the $PI$ based on varying inlet velocities and the probability of infection across different areas. Furthermore, we improved the equation of the $PI$ to ensure accurate assessment of the probability of infection on page11, lines 309-312.

Regarding the potential typo in the legend, the vertical axis in the bar chart is represented in scientific notation instead of percentages. We mistakenly labeled the units on the vertical axis in the legend as percentages, and we have corrected it. Additionally, we have included the values of $PI$ at different inlet speeds in Figure 13, contrasting them with the probability of infection, to collectively summarize the variations in infection probability under different circumstances.
