# Supplementary material for: Optimizing cabin air inlet velocities and personal risk assessment: Introducing the Personal Contamination Ratio (PCR) method for enhanced aircraft cabin infection risk evaluation
Source: PLoS One. 2024 Sep 6;19(9):e0309730. doi: 10.1371/journal.pone.0309730 (PMC11379313; doi:10.1371/journal.pone.0309730)
Supplement: Attachment — Submitted filename: Response to Reviewers_v6.docx [file pone.0309730.s005.docx]

**Ref:** PONE-D-24-00989

**Title:** Optimizing cabin air inlet velocities and personal risk assessment: introducing the Personal Contamination Ratio (PCR) method for enhanced aircraft cabin infection risk evaluation

Dear Editor and Reviewers,

We greatly appreciate your valuable comments and suggestions which have helped enhance our manuscript. Based on your feedback, we have made improvements to our work. We have addressed each of your points in detail, with changes highlighted in red in the revised manuscript and responses marked in blue below.

**Reviewer #3's Comments**

The authors provide references to sources providing information about the conditions representing the tested case.

The authors provided drawings and data explaining the adopted methods and results to demonstrate the usefulness of the $PCR$ method.

This study contributes important information on droplet transport dynamics and infection risk in aircraft cabins, highlighting the importance of optimizing air delivery rates. $PCR$ can significantly contribute to better design of ventilation systems and public health strategies.

We are truly grateful for the reviewer's supportive feedback, which means a lot to us. We appreciate the time and effort invested in reviewing our manuscript, and we are pleased that the revisions have met with approval. Thank you for recognizing the value of our research and for your encouragement.

**Reviewer #4's Comments**

The authors would like to express their sincere gratitude to the reviewer for providing thoughtful and valuable feedback on this manuscript. The improvements made to the manuscript were greatly influenced by the reviewer's insightful comments.

The manuscript entitled “Optimizing cabin air inlet velocities and personal risk assessment: introducing the Personal Contamination Ratio ($PCR$) method for enhanced aircraft cabin infection risk evaluation” is interesting. However, there are several major concerns that authors shall address.

1.Abstract: Measurable findings are required in the abstract session. Conclusion Is not clearly highlighted as in current form.

Response: We appreciate the reviewer for reminding this issue. Based on the reviewer’s comment, we have revised the content of the abstract section as follows:

*‘Recurrent epidemics of respiratory infections have drawn attention from the academic community and the general public in recent years. Aircraft plays a pivotal role in facilitating the cross-regional transmission of pathogens. In this study, we initially utilized an Airbus A320 model for computational fluid dynamics (CFD) simulations, subsequently validating the model's efficacy in characterizing cabin airflow patterns through comparison with empirical data. Building upon this validated framework, we investigate the transport dynamics of droplets of varying sizes under three air supply velocities. The Euler-Lagrangian method is employed to meticulously track key parameters associated with droplet transport. This study integrates acquired data into a novel PCR (Personal Contamination Rate) equation to assess individual contamination rates. Numerical simulations demonstrate that increasing air supply velocity leads to enhanced stability in the movement of larger particles compared to smaller ones. Results show that the number of potential infections in the cabin decreases by 51.8 % at the highest air supply velocity compared to the base air supply velocity, and the total exposure risk rate reduced by 26.4 %. Thus, optimizing air supply velocity within a specific range effectively reduces the potential infection area. In contrast to previous research, this study provides a more comprehensive analysis of droplet movement dynamics across various particle sizes. We introduce an improved method for calculating the breathing zone, thereby enhancing droplet counting accuracy. It is hoped that these findings can provide valuable insights for enhancing non-pharmacological public health interventions and improving cabin ventilation system design.’*

Please see Page 1, lines 11-29 in the revised manuscript.

Additionally, we have also revised the content of the conclusion section as follows:

*‘(1) The direction of airflow within the aircraft cabin is closely linked to the inlet velocity, a critical parameter in cabin ventilation systems. Our investigation reveals that air supplied through the ventilation systems converges near the lower section of the aisle before ascending, regardless of the inlet velocity. This airflow pattern is significantly influenced by thermal plumes generated by human occupants and their respiratory processes. Notably, as the inlet velocity increases, the rearward flow of air becomes more pronounced. These observations underscore the complex interplay between inlet velocity, thermal plumes, and the resultant airflow patterns within the cabin environment.*

*(2) The trajectories of small-sized droplets exhibit high sensitivity to variations in inlet air velocity. As the inlet air supply incrementally increases from its minimum threshold, these droplets demonstrate a tendency to converge, resulting in a relative reduction of potential infection areas. In contrast, larger droplets display a slower response to changes in inlet air velocity. Their trajectories undergo significant alterations only in response to substantial increases in inlet air velocity.*

*(3) Optimizing the inlet air velocity is crucial for effective infection control. Experimental results demonstrate that the minimum inlet velocity leads to the largest potential infection area. Conversely, maximum velocity reduces the potential infection area in the transverse direction but significantly increases it longitudinally. Notably, the number of potential infections within the cabin at the highest inlet air velocity decreases by 51.8 % compared to the basic inlet air velocity, and the total exposure risk rate is reduced by 26 %. Furthermore, the number of rows with infected passengers decreases to 4 rows under maximum inlet air velocity. The probability of infection for more than 50% of individuals is reduced by 65.2 % at the highest inlet air velocity and by 21.74 % at moderate inlet air velocity compared to the basic inlet air velocity. Additionally, the total number of individuals at risk of infection is nearly halved at the highest inlet air velocity. Considering passenger comfort alongside infection control, operating at a moderate velocity emerges as the optimal strategy. This balanced approach effectively mitigates infection risk while maintaining acceptable comfort levels for passengers. In this study, only one pathogen-releasing CSP, seated in position 5C, was considered.’*

Please see Page 17, lines 488-502; Page 18, lines 503-515 in the revised manuscript.

2. Introduction- suggest adding on other airborne infection statistics and its description, instead of COVID-19 only. These inclusions could show the importance of present study to be adopted in future.

Response: Thanks for bring up this issue. We have searched the statistical data on various respiratory diseases and have provided a specific example for context. The details of the revision are as follows:

*‘Airborne infectious diseases pose a significant threat to global public health, with the potential to spread rapidly and overwhelm healthcare systems during outbreaks. These respiratory-borne illnesses can lead to widespread infections, affecting millions of people worldwide each year. The transmission of such diseases has become a focal point of research, particularly in light of recent global health crises. Among the various airborne infectious diseases, some stand out due to their impact and prevalence. For instance, Respiratory Syncytial Virus (RSV) spreads extensively during certain periods each year, with a median duration of 4.6 months, affecting millions globally [1]. More recently, the COVID-19 pandemic has had an unprecedented global impact, resulting in 775.69 million confirmed cases and 6.95 million deaths. This pandemic has significantly raised awareness about the transmission of respiratory diseases and led to extensive research and vaccination campaigns. The emergence of influenza during the later stages of the COVID-19 pandemic further highlights the threat posed by respiratory diseases. These outbreaks have intensified the focus on transmission studies, revealing the potential for rapid spread and severe strain on healthcare resources. In the current globalization period, air travel in particular has become an essential way of linking people worldwide. In this mode of transportation, air cabin plays an important role in spreading infectious diseases.’*

Please see Page 2, lines 32-47 in the revised manuscript.

Additionally, to support our findings, we have included relevant literature reference, which is listed below:

[1] Agca H, Akalin H, Saglik I, Hacimustafaoglu M, Celebi S, Ener B. Changing epidemiology of influenza and other respiratory viruses in the first year of COVID-19 pandemic. Journal of Infection and Public Health. 2021;14(9):1186-90.

3. Description of droplets shall be included, i.e., material, density, viscosity, other physical properties.

Response: Thank you for bringing this important issue to our attention. We agree that it is crucial for accurate simulation. Based on equivalent aerodynamic principles, this approach simplifies and predicts the motion, settling, and spreading behavior of real particles or droplets by comparing them to idealized spherical particles with the same aerodynamic properties. In our study, we defined droplets as inert particles and modeled them with a density of $1000 kg/m^{3}$ to reflect their water-liquid composition. Based on the reviewer’s comment, we have added a detailed description of the droplets as follows:

*‘**To simulate the droplets based on equivalent aerodynamic principles, we defined them as inert particles, allowing us to focus on their aerodynamic behavior and physical characteristics without involving complex chemical reactions or biodegradation processes. Based on previous research, the droplets are modeled using water-liquid, with a density of* $1000 kg/m^{3}$*. This configuration ensures that we can accurately capture the droplets’ behavior in the airflow.’*

Please see Page 8, lines 226-230 in the revised manuscript.

Furthermore, to support the configuration used in our study, we have provided relevant literature references as listed below. These references also set the droplets as inert particles, and the same physical properties as in our study. In addition, due to the low-humidity nature of the cabin environment during flight, our previous research demonstrated that droplets would rapidly evaporate into a residual [2-4], which is expected to minimize their effect on droplet transport characteristics. However, since this study modeled a large-scale, densely populated cabin environment and considering the high computational resources required, evaporation was temporarily not considered.

[2] Li P, Liu W, Zhang TT. CFD modeling of dynamic airflow and particle transmission in an aircraft lavatory. Building Simulation. 2023;16(8):1375-90.

[3] Ma B, Ruwet V, Corieri P, Theunissen R, Riethmuller M, Darquenne C. CFD simulation and experimental validation of fluid flow and particle transport in a model of alveolated airways. Journal of Aerosol Science. 2009;40(5):403-14.

[4] Park CIP, editor Simulating aerosol movement in experimental chambers using computational fluid dynamics. CSBE/SCGAB 2017 Annual Conference; 2017.

4. Is humidity being considered in this study? If no, please justify thoroughly. As far as reviewer concern, humidity could significantly affect the droplets transportation characteristics.

Response: Thank you for your insightful question regarding the consideration of humidity in our study. We appreciate your concern about the potential impact of humidity on droplet transport characteristics. In this study, we did not explicitly consider humidity as an important factor. Our decision was based on several considerations. Firstly, our previous research has shown that in the low humidity environment of an aircraft cabin, droplets tend to evaporate rapidly after emission. For example, droplets with a diameter of 10 $\mu m$ typically complete the evaporation process in less than 0.1 seconds. Notably, the smaller the droplet, the faster the evaporation process. This quick evaporation process minimizes the influence of environmental humidity on droplet transport [5-7].

Moreover, the primary focus of our research was to introduce the $PCR$ (Personal Contamination Ratio) method. Given this emphasis, we chose to simplify certain environmental parameters in our simulation phase to highlight the efficacy of the $PCR$ approach.

However, we acknowledge the importance of humidity in this filed study. Additionally, we included the following limitation section in the manuscript. Furthermore, considering that accounting for evaporation effects in the cabin environment would significantly increase computational cost, we have decided, based on our previous studies, to disregard the evaporation of droplets. Rather, we shall concentrate our research on developing the $PCR$ method.

*‘In this study, we did not explicitly account for the effects of humidity on droplet transport within the aircraft cabin environment. Our decision was informed by previous research indicating that droplets tend to evaporate rapidly in the extremely dry conditions of an aircraft cabin, thereby minimizing the impact of ambient humidity on droplet dynamics. This simplification was necessary to focus on the primary objective of our research: the development and validation of the Personal Contamination Ratio (*$PCR$*) method. While this approach allowed us to highlight the efficacy of the* $PCR$ *method, we recognize that excluding humidity and evaporation effects may limit the generalizability of our findings to other environments. Future studies should consider more factors to provide a more comprehensive understanding of droplet behavior under varying humidity conditions.’*

Please see Page 18, lines 520-529 in the revised manuscript.

[5] Shang Y, Dong J, Tian L, He F, Tu J. An improved numerical model for epidemic transmission and infection risks assessment in indoor environment. Journal of Aerosol Science. 2022 May 1;162:105943.

[6] Li X, Shang Y, Yan Y, Yang L, Tu J. Modelling of evaporation of cough droplets in inhomogeneous humidity fields using the multi-component Eulerian-Lagrangian approach. Building and Environment. 2018 Jan 15;128:68-76.

[7] Li X, Yan Y, Fang X, Tu J. Numerical studies of indoor particulate and gaseous micropollutant transport and its impact on human health in densely-occupied spaces. Environmental Pollution. 2024 Feb 1;342:123031.

5. Authors shall justify why SIMPLE scheme for pressure velocity coupling and second order upwind scheme are chosen. Else, authors might need to find reference (similar study that investigate the effect of droplets dispersion in indoor) to support. Example: <https://doi.org/10.1016/j.enbuild.2023.113439>

Response: We greatly appreciate your valuable question regarding our choice of the SIMPLE scheme for pressure-velocity coupling and the second-order upwind scheme. Furthermore, it has been demonstrated in earlier research that the second-order upwind scheme and the SIMPLE method for pressure-velocity coupling are especially well-suited for simulations of interior environments.

Following your suggestion, we have revised the description of the SIMPLE scheme as follows:

*‘This study employs the SIMPLE scheme for pressure-velocity coupling and the second-order upwind scheme for momentum space discretization. This combination has been proven particularly suitable for indoor environment simulations, aligning well with the scope of our study [8,9].’*

Please see Page 7, line 209 and Page 8, lines 210-211 in the revised manuscript.

The authors have carefully read the literature provided by the reviewers and it was found the literature provide valuable insight to our manuscript, and we have added the following references in the manuscript:

[8] Tan H, Othman MHD, Kek HY, Chong WT, Wong SL, Ern GKP, et al. Would sneezing increase the risk of passengers contracting airborne infection? A validated numerical assessment in a public elevator. Energy and Buildings. 2023;297:113439.

[9] Ho X, Ho WS, Wong KY, Hassim MH, Hashim H, Ab Muis Z, et al. Study of fresh air supply vent on indoor airflow and energy consumption in an enclosed space. 2021;83.

These studies employed numerical methods similar to ours and achieved reliable results in simulating indoor droplet dispersion, providing strong support for our methodological choices.

6. Line 340- satisfactory agreement could be subjective. What is the relative error? This information is very crucial to support the reliability of result.

Response: Thank you for your insightful comment. Regarding your query on Line 340, the figure in question compares the overall direction of the experimental and simulated flow fields. Our objective was to demonstrate that the general trends align, rather than evaluating the difference at each individual value. While we acknowledge that a relative error analysis could provide further detail, the focus of this figure is to illustrate the broader agreement in flow patterns.

The intent is to show that the simulated flow field accurately captures the overall behavior observed in the experimental data, which we believe sufficiently the reliability of our results. The broader agreement in flow patterns indicates that the simulation model is robust and capable of replicating the key characteristics of the experimental flow field. Therefore, we consider the general trend alignment to be a satisfactory demonstration of the model validation, and the relative error at each point, while informative, is not critical for this particular comparison.

To further substantiate our approach, we refer to several studies that have adopted a similar methodology, focusing on overall trend alignment to validate simulation models as follows:

[10] Yan Y, Li X, Shang Y, Tu J. Evaluation of airborne disease infection risks in an airliner cabin using the Lagrangian-based Wells-Riley approach. Building and environment. 2017 Aug 15;121:79-92.

[11] Yan Y, Li X, Yang L, Yan P, Tu J. Evaluation of cough-jet effects on the transport characteristics of respiratory-induced contaminants in airline passengers’ local environments. Building and environment. 2020 Oct 1;183:107206.

[12] Liu M, Liu J, Cao Q, Li X, Liu S, Ji S, Lin CH, Wei D, Shen X, Long Z, Chen Q. Evaluation of different air distribution systems in a commercial airliner cabin in terms of comfort and COVID-19 infection risk. Building and Environment. 2022 Jan 15;208:108590.

[13] Li J, Cao X, Liu J, Wang C, Zhang Y. Global airflow field distribution in a cabin mock-up measured via large-scale 2D-PIV. Building and Environment. 2015 Nov 1;93:234-44.
